# Dataset on the superabsorbent hydrogel synthesis with SiO_2_ nanoparticle and role in water restoration capability of agriculture soil

**DOI:** 10.1016/j.dib.2017.05.046

**Published:** 2017-05-31

**Authors:** Vinay Mohan Pathak, Navneet Kumar

**Affiliations:** Department of Botany & Microbiology, Gurukul Kangri University, Haridwar, 249404 Uttarakhand, India

**Keywords:** Biodegradation, Nanoparticles, Polymer, Hydrogel

## Abstract

Synthetic polymer was exploited as water-superabsorbent hydrogel and helped to conserve water in the agricultural soil. The hydrogel polymers were synthesized the carboxymethyl cellulose (CMC) and starch in addition to SiO_2_ nanoparticles. Superabsorbent hydrogel polymer having 35% water retention ability was analyzed with three replicates. Hydrogel increased the water restoration capability of agricultural soil.

## **Specifications Table**

**Table:** 

Subject area	Agronomy, Ecology, Nanotechnology
More specific subject area	Exploitation of nanoparticles in hydrogel formation and their effect on water conservation capability
Type of data	Tables, Figures, Text file
How data was acquired	Synthesis of superabsorbent hydrogel with SiO_2_ nanoparticles;
	Electrical conductivity of polymer film was analyzed;
	Hydrogel polymer was mixed in agriculture soil
Data format	Raw, Analyzed
Experimental factors	Role of nanoparticles in hydrogel formation and their effects on water restoration capability.
Experimental features	The relationship between the addition of nanoparticles on hydrogel formation and water holding ability
Data accessibility	The data are available with this article

## **Value of the data**

• This data could be used as water restoration tool in agriculture field that is deficient with water.
• This data will also help in developing eco-friendly polymer to reduce the mulching film problems in agricultural practices.
• This data represented the impact of nanoparticles on the synthesis of superabsorbent hydrogel polymer.

## 1. Data

**Fig. 1:**
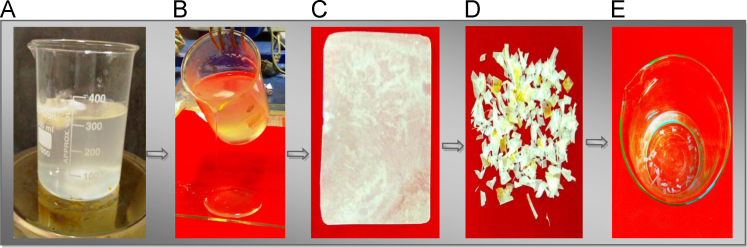
A–C showing the synthesis of polymer film, D–E pieces of polymer film and formation of water absorbent hydrogel.

**Fig. 2:**
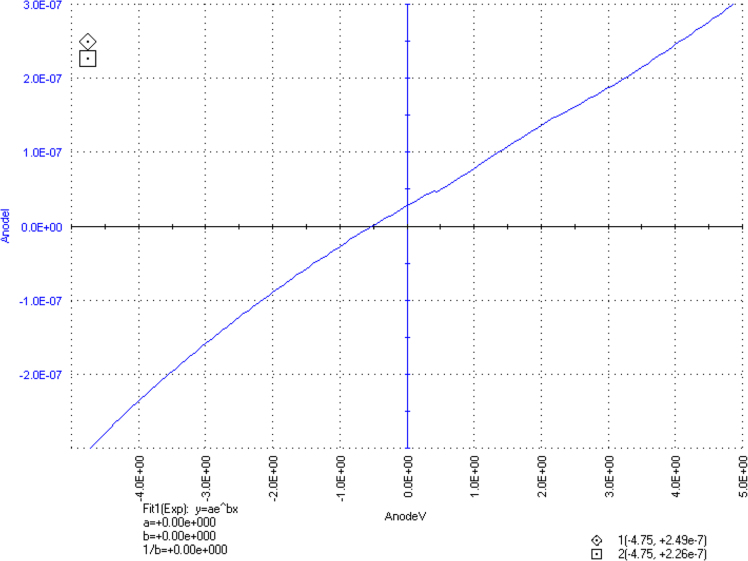
Electrical conductivity of polymer film.

**Fig. 3:**
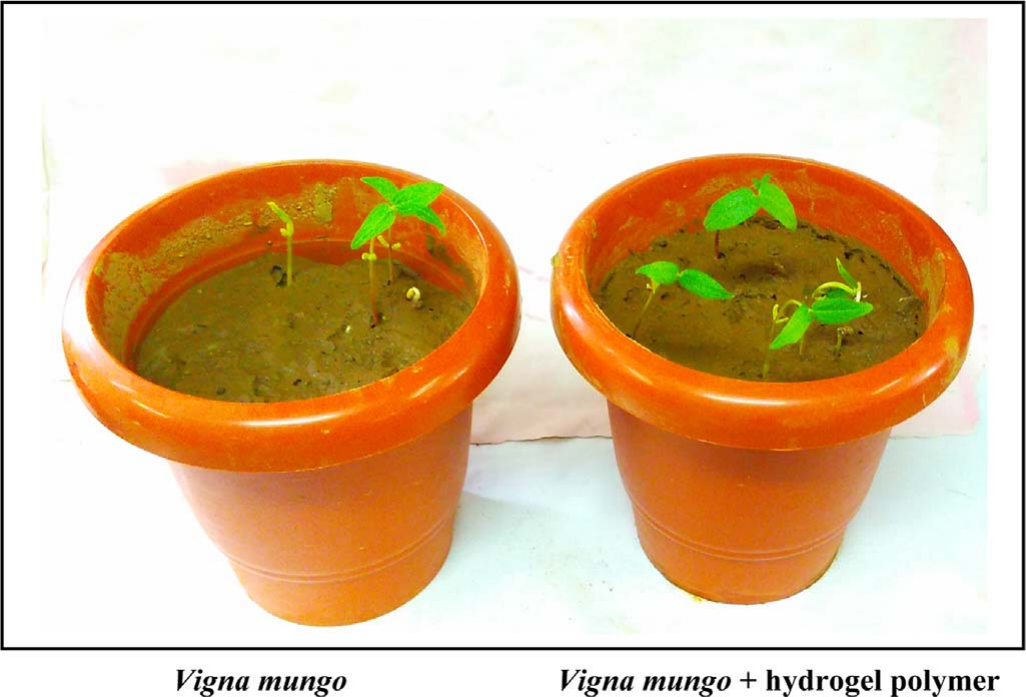
Growth of *Vigna mungo* plants in hydrogel amended soil at day 10.

The dataset of this article described the consequence of SiO_2_ nanoparticles in the synthesis of superabsorbent hydrogel polymer from the crosslinking in between carboxymethyl cellulose (CMC) and starch with aluminum sulfate. The Fig. 1 shows the polymer film formation and hydrogel, Fig. 2 shows the electrical conductivity property of polymer film, Fig. 3 shows the plant growth. Table 1 shows the water retention of hydrogel with differing concentration of aluminium sulfate and Table 2 shows the growth of the plant in the presence and absence of hydrogel polymer.

**Table 1:** Water retention of hydrogel with differ concentration of aluminum sulfate.

**S. no.**	**Aluminum sulfate by weight (%)**	**Water retention (g/g)**
1	0	7.78
2	1	18.72
3	1.5	27.3
4	2	35.73
5	2.5	29.01
6	3	19.24
7	3.5	11.17

**Table 2:** Germination and growth of *Vigna mungo* in the absence and presence of superabsorbent hydrogel polymer.

**S. no**	**Germination/growth**	***Vigna mungo***	***Vigna mungo*****+hydrogel polymer**
1	Germination (%)	50	60
2	Shoot height (cm)	9.5±0.55	10.7±05
3	Leaf length (cm)	2.4±0.45	2.73±0.47
4	Root length	6.23±0.45	6.46±0.35

## 2 Experimental design, materials and methods:

Synthesis of hydrogel polymer was conducted with SiO_2_ nanoparticles and analyzed for water retention capacity. Carboxymethyl cellulose sodium salt (CMC) and starch soluble in addition to SiO_2_ (20 nm) nanoparticles (enhance the surface area) were used to synthesize superabsorbent polymer films and aluminum sulfate octadecahydrate was used to establish crosslink in between polymer composite [4], [5].The DC electrical conductivities of polymer samples were analyzed by using 4200-SCS Keithley, it is a modular uses for characterization of electrical properties of materials. Two metallic electrodes were then connected to samples using silver wires. The transient I–V measurements done at room temperature. I–V characteristics help to determine electrical conductivities polymer samples. The amount of water retention was calculated by the formula (Gs−Gi)/Gi, where Gs is the weight of hydrogel after water absorption and Gi is the original weight of the superabsorbent polymer. Superabsorbent polymer was mixed with agriculture soil and used for sowing seeds of *Vigna mungo* [1], [2], [3], [4], [5]. Three replicates were used for the investigation of plants growth and germination of seeds.
