# Supplementary material for: Dataset on the superabsorbent hydrogel synthesis with SiO2 nanoparticle and role in water restoration capability of agriculture soil
Source: Data Brief. 2017 May 31;13:291–4. doi: 10.1016/j.dib.2017.05.046 (PMC5470529; doi:10.1016/j.dib.2017.05.046)
Supplement: Supplementary file 1 — Supplementary material [file mmc1.docx]

**Competing interests:**

The authors declare that they have no competing interest.
